# Sustainable food security in India—Domestic production and macronutrient availability

**DOI:** 10.1371/journal.pone.0193766

**Published:** 2018-03-23

**Authors:** Hannah Ritchie, David Reay, Peter Higgins

**Affiliations:** 1 School of Geosciences, University of Edinburgh, Edinburgh, United Kingdom; 2 Moray House School of Education, University of Edinburgh, Edinburgh, United Kingdom; College of Agricultural Sciences, UNITED STATES

## Abstract

India has been perceived as a development enigma: Recent rates of economic growth have not been matched by similar rates in health and nutritional improvements. To meet the second Sustainable Development Goal (SDG2) of achieving zero hunger by 2030, India faces a substantial challenge in meeting basic nutritional needs in addition to addressing population, environmental and dietary pressures. Here we have mapped—for the first time—the Indian food system from crop production to household-level availability across three key macronutrients categories of ‘calories’, ‘digestible protein’ and ‘fat’. To better understand the potential of reduced food chain losses and improved crop yields to close future food deficits, scenario analysis was conducted to 2030 and 2050. Under India’s current self-sufficiency model, our analysis indicates severe shortfalls in availability of all macronutrients across a large proportion (>60%) of the Indian population. The extent of projected shortfalls continues to grow such that, even in ambitious waste reduction and yield scenarios, enhanced domestic production alone will be inadequate in closing the nutrition supply gap. We suggest that to meet SDG2 India will need to take a combined approach of optimising domestic production and increasing its participation in global trade.

## Introduction

In 2015, the United Nations (UN) committed to achieving zero hunger by 2030 as the second of the Sustainable Development Goals (SDGs). An important element of this goal is to end all forms of malnutrition, including agreed targets on childhood stunting and wasting. This represents an important progression beyond the Millennium Development Goals (MDGs), where food security was defined and measured solely on the basis of basic energy requirements (caloric intake), and prevalence of underweight children [1]. This new commitment has significant implications for the focus of research and policy decisions; it requires a broadening of scope beyond the traditional analysis of energy intake, and inclusion of all nutrients necessary for adequate nourishment.

India offers a potentially unique example in the development of models and mechanisms by which nutritional needs can be addressed sustainably. In 2016, India ranked 97 out of 118 on the Global Hunger Index (GHI)—this rates nations’ nutritional status based on indicators of undernourishment, child wasting, stunting and mortality [2]. Despite ranking above some of the world’s poorest nations, India’s reduction in malnourishment has been slow relative to its recent strong economic growth and puts it behind poorer neighbouring countries [3]; India has fallen from 80^th^ to 97^th^ since 2000.

India’s nutritional problems are extensive. In 2016, 38.7% of children under five were defined as ‘stunted’ (of below average height) [2], a strong indicator of chronic malnourishment in children and pregnant women, and a largely irreversible condition leading to reduced physical and mental development [4]. Malnourishment within the adult population is also severe, with approximately 15% of the total population defined as malnourished. The issue of malnutrition in India is complex, and determined by a combination of dietary intake and diversity, disease burden (intensified by poor sanitation and hygiene standards), and female empowerment and education [5]. Improvements in dietary intake alone will therefore by insufficient to eliminate malnutrition, however it forms an integral component alongside progress in other social and health indicators—particularly sanitation. Quantification of India’s micronutrient and amino acid profiles, and recommendations for addressing these deficiencies have been completed as a follow-up paper (Ritchie et al. in submission) to provide a more holistic overview of its nutritional position.

India’s nutritional and health challenges are likely to be compounded in the coming decades through population growth and resource pressures. Its current population of 1.26 billion is projected to increase to 1.6 billion by 2050, overtaking China as the world’s most populous nation [6]. India has also been highlighted as one of the most risk-prone nations for climate change impacts, water scarcity, and declining soil fertility through land degradation [7].

A number of studies have focused specifically on Indian food intake and malnutrition issues from survey assessments at the household level [8]. The emphasis within India’s agricultural policy and assessment of its success has traditionally been on energy (caloric) intake [9]. Since the Green Revolution in the 1970s, agricultural policies have been oriented towards a rapid increase in the production of high-yielding cereal crops with a focus to meet the basic calorific needs of a growing population. India has attempted to reach self-sufficiency predominantly through political and investment orientation towards wheat and rice varieties [10]. While production of staple crops has increased significantly, India’s agricultural policy focus on cereal production raises a key challenge in simultaneously meeting nutritional needs in caloric, high-quality protein and fat intakes. Few studies have addressed the system-wide balance between supply and demand of the three key macronutrients—calories, protein and fat; nor have they assessed the importance of protein quality through digestibility and amino acid scoring. This assessment is particularly significant for India as a result of its extensive and complex malnutrition issues. Whether India is capable of meeting these macronutrient needs in the future through domestic production improvements alone is of prime importance for study, as a result of its growing population and policy orientation towards self-sufficiency.

Improving the availability and access to food at the consumer level requires an understanding of how food is created and lost through its various pathways across the full agricultural supply chain. Here, for the first time, we have attempted to capture this high-level outlook from crop harvesting to residual food availability across the three macronutrient categories.

## Methods

### Mapping the current Indian food system

The Indian food system was mapped from crop production through to per capita food supply using FAO Food Balance Sheets (FBS) from its FAOstats databases [11]. FBS provide quantitative data (by mass) on production of food items and primary commodities, and their utilisations throughout the food supply chain. Such data are available at national, regional and global levels. Food Balance Sheet data for 2011 have been used, these being from the latest full data-set available. Some aspects of FBS data are available for the years 2012 and 2013, however such data are not complete across all commodities and value chain stages at the time of writing.

Food Balance Sheets provide mass quantities across the following stages of the supply chain: crop production, exports, imports, stock variation, re-sown produce, animal feed, other non-food uses, and food supplied (as kg per capita per year). Data on all key food items and commodities across all food groups (cereals; roots and tubers; oilseeds and pulses; fruit and vegetables; fish and seafood; and meat and dairy) are included within these balances.

While there are uncertainties in FAO data (see Supplementary Information for further discussion on FAO data limitations), FBS provide the only complete dataset available for full commodity chain analysis. Therefore, while not perfect, they provide an invaluable high-level outlook of relative contribution of each stage in the food production and distribution system. As shown in this study (see Results section below), a top-down model using FAO FBS has a discrepancy of <10% with national nutrition survey results at the household level.

FBS do not provide food loss and waste figures by stage in the supply chain. To maintain consistency with FAO literature, food loss figures have therefore been calculated based on South Asian regional percentages within FAO publications [12]. These percentage figures break food losses down across seven commodity groups and five supply chain stages (agricultural production, postharvest handling and storage, processing and packaging, distribution and consumption). The applied percentage values by commodity type and supply chain stage are provided in S1 Table.

In order to calculate the total nutritional value at each supply chain stage, commodity mass quantities were multiplied by FAO macronutrient nutritional factors [11]. In this analysis, energy content (kilocalories), protein, and fat supply were analysed. Protein quality is a key concern for India in particular as a result of its largely grain-based diet, with grains tending to have poorer digestibility and amino acid (AA) profiles than animal-based products and plant-based legume alternatives [13]. To best quantify limitations in protein quality in the Indian diet, protein intakes have therefore been corrected for digestibility using FAO digestibility values [14].

For consistency, and to provide a better understanding of the food system down to the individual supply level, all metrics have been normalised to average per person per day (pppd) availability using UN population figures and prospects data [6]. Whilst this provides an average per capita availability value, it does not account for variability in actual macronutrient supply within the population. To help adjust for this, we have also estimated the assumed distribution of supply of each macronutrient using the FAO’s preferred log-normal distribution and India-specific coefficient variation (CV) factor of 0.26 [15]. Whilst we recognise that food requirements vary between demographics based on age, gender and activity levels, the normalisation of food units to average per capita supply levels is essential in providing relatable measures of food losses within the system, and its measure relative to demographically-weighted average nutritional requirements (as described below) is appropriate in providing an estimation of the risk of malnourishment.

Estimated macronutrient supply has then been compared to recommended intake values. The FAO defines the “Average Daily Energy Requirement” (ADER)—for India’s demographic specifically—as 2269kcal pppd; ADER is defined as the average caloric intake necessary to maintain a healthy weight based on the demographics, occupation, and activity levels of any given population [16]. Protein requirements can vary between similar individuals; recommended daily amounts (RDA) are therefore typically given as two standard deviations (SD) above the average requirement to provide a safety margin, which some individuals would be at risk of falling below. The World Health Organization (WHO) define a ‘safe’ (recommended) intake in adults of 0.83 grams per kilogram per day (g/kg/d) of body mass for proteins with a digestibility score of 1.0 [17]. The average vegetarian Indian diet contains lower intakes of animal-based complete proteins; the Indian Institute of Nutrition therefore recommends a higher intake of 1 g/kg/d of total protein for Indians to ensure requirements of high-quality protein are met [18]. This is equivalent to 55 and 60 grams of protein per day in average adult females and males, respectively based on mean body weight [19]. Since our analysis attempts to correct for protein digestibility, WHO’s lower safe intake of 0.83g/kg/d would reduce to an equivalent of 50 grams of high-quality protein per day for an average 60 kilogram individual. Consequently in this study we have adopted this RDA value of 50 gpppd.

Dietary fat intake plays a key dietary role in the absorption of essential micronutrients. Several vital vitamins, including vitamin A, D, E and K are fat-soluble—insufficient intake can therefore result in poor micronutrient absorption and utilisation [20]. Inadequate fat intake can therefore exacerbate the widespread ‘hidden hunger’ (micronutrient deficiency) challenge in India [21] through poor nutrient absorption. However, daily requirements for fatty acids are less straightforward to determine, relative to energy or protein—there is no widely-agreed figure for total fat requirements for adequate nutrition [22]. The resolution of food balance sheet data does not allow us to adequately quantity the availability to the level of specific fatty acids. As a result, although we have mapped pathways of total fat availability through the food system in a similar manner to energy and protein, we have not here attempted to quantity the prevalence of potential insufficiency at the household level.

### Mapping potential near-term and long-term scenarios

Our initial analysis identified two mechanisms potentially crucial in increasing food availability at the household level: reduction of harvesting, postharvest and distribution losses; and improvements in crop yields. Medium-term (through to 2030) and long-term (2050) scenarios have therefore been mapped based on use of these mechanisms. It should be noted that these scenarios are focused on domestic **supply-side** measures to enhance food availability as opposed to demand drivers related to consumer preferences. A summary of assumptions used in each scenario in this analysis is provided in S2 Table.

A 2030 baseline scenario (assuming yields stagnate and population growth continues in line with UN projections) and three alternative scenarios to 2030 were analysed:

**Scenario 1 (halving food supply chain losses):** it was assumed that a significant shift in post-harvest management practices, appropriate refrigeration, and efficient distribution allowed for a halving of food loss percentages at the production, postharvest, processing and distribution stages of the supply chain. This would make its relative losses more in line with those of more developed nations [12]. In this scenario consumption (household) waste was assumed to remain constant.

**Scenario 2 (achieving 50% of attainable yield (AY) across all key crops):** the halving of food chain losses in scenario 1 was assumed. In addition, it was assumed that all key crops managed to achieve 50% AY through better agricultural management, irrigation and fertiliser practices. ‘Attainable yield’ is defined as the **yield** achieved with best management practices including pest, nutrient (i.e. nutrients are not limiting) and water management.

**Scenario 3 (achieving 75% AY across all key crops):** assumptions as in scenario 2 except an attainment of 75%, rather than 50% AY, has been assumed through crop yield improvements.

Long-term (through to 2050) scenarios were as follows:

**Scenario 1 (halving food supply chain losses):** the same assumption of halving food loss percentages at the production, postharvest, processing and distribution stages of the supply chain was applied in this scenario. This will require a significant shift in post-harvest management practices, appropriate refrigeration, and efficient distribution, hence 50% reduction represents a magnitude which is more likely to be achieved in this long-term scenario than in the near-term.

**Scenario 2 (achieving 75% AY across all key crops):** the same assumption of a closure of the yield gap to 75% AY across all crop types, as in the near-term scenario 3, was applied.

**Scenario 3 (achieving 90% AY across all key crops):** it was assumed that all crop types managed to achieve closure of the yield gap to 90% AY.

To correct for 2030 and 2050 population estimates, all metrics were re-normalised to ‘per person per day’ (pppd) based on a projected Indian population estimate from UN prospects medium fertility scenarios [6].

To best demonstrate the food production potential of current agricultural support mechanisms, such as governmental policy and subsidy (which largely determine crop choices), the relative allocation of crop production was assumed constant. It was also assumed that production increases were achieved through agricultural intensification alone; this assumption was based on FAOstats data which has shown no increase in agricultural land area over the past decade, indicating a stagnation in agricultural extensification (http://faostat.fao.org/beta/en/#home).

Crop yield increases were derived based on closure of current farm yields (FY) to reported attainable yields (AY). FY is defined as the average on-farm yield achieved by farmers within a given region, and AY is defined as the economically attainable (optimal) yield which could be achieved if best practices in water and pest management, fertiliser application and technologies are utilised in non-nutrient limiting conditions). Estimates of crop yield improvements were based on given percentage realisations of maximum attainable yields (AY) attained from published Indian crop-specific figures [23]. These data are available across all key crop types. Baseline and AY values are provided in S3 Table.

Significant improvements in yield would predominantly be achieved through improved nutrient and water management. In the present study, scenarios were mapped based on achievement of 50% and 75% AY in the near-term. Fifty percent AY should be technically feasible by 2030: many crops have already reached these values, and those which have yet to do so, typically fall short by 3–5% (see S3 Table for baseline, and AY values). Attainment of 75% AY would be highly ambitious in the near-term, representing an increase of >20% in yield. However, 75% AY and higher may be feasible in the long-term if significant investment in agricultural management and best practice were to be realised in this sector.

Our scenarios to 2050 are therefore modelled on the basis of closure of the yield gap to 75% and 90% AY. To assess whether these estimates were realistic, necessary growth rates were cross-checked based on historical yield growth rates in India. Discussion on this comparison and the suitability of attainable yield valuables utilised in this study are available in the Supplementary Discussion.

Climate change impacts on crop yields remain highly uncertain; the importance of temperature thresholds in overall crop tolerance makes yield impacts highly dependent on GHG emission scenarios. This makes it challenging to accurately quantify 2050 climate impacts. As such, we applied average percentage changes in yields of Indian staple crops based on literature review [24] of field-based observations and climate model results. The studies utilised presented results for a doubling of atmospheric CO_2_ from pre-industrial levels. This approximates to a business-as-usual (BAU) scenario for 2050 [25]. The yield-climate factors applied in this analysis are provided in S4 Table.

It is projected that, through economic growth and shifts in dietary preferences, meat and dairy demand in India will continue to increase through to 2050. It has been assumed that per capita demand in 2050 is in line with FAO projections; this represents an increase in meat from 3.1kg per person per year (2007) to 18.3kg in 2050, and an increase in milk and dairy from 67kg to 110kg per person per year [26]. We here assume that this increase in livestock production has been met through increased production of crop-based animal feed rather than pasture. The change in macronutrient demand for animal feed was calculated based on energy and protein conversion efficiency factors for dominant livestock types (beef cattle, dairy cattle, ruminants and poultry) [27].

Our analysis assumes that the per person allocation of crops for resowing and non-food uses, and the relative allocation of land for respective crop selection, is the same as in the initial baseline (2011) analysis.

## Results

### Current food system pathways

**Fig 1:**
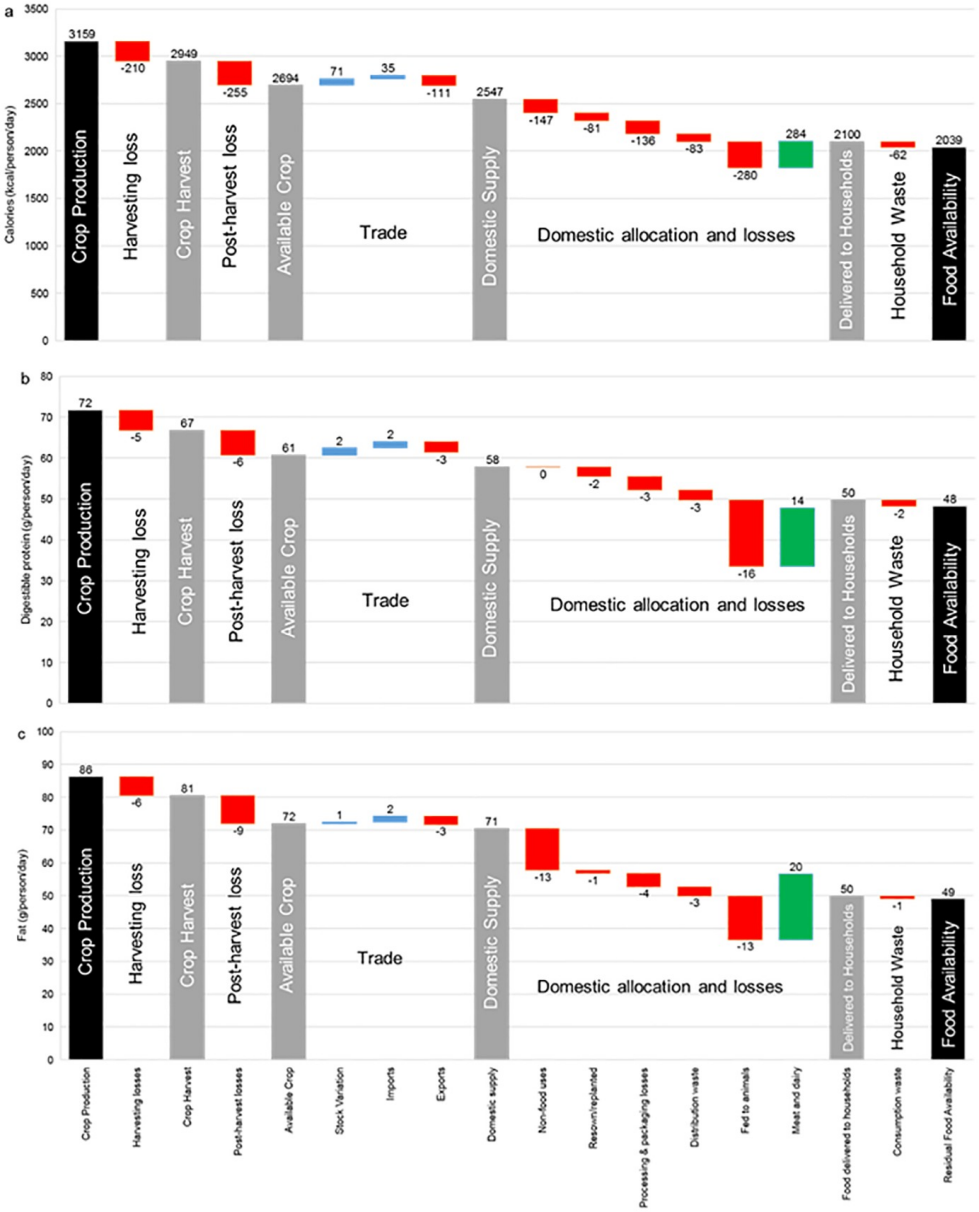
Production and losses in the Indian food system from ‘field to fork’ in 2011. Food pathways in (a) calories; (b) digestible protein; and (c) fat from crop production to residual food availability, normalised to average per capita levels assuming equal distribution. Red bars (negative numbers) indicate food system losses; blue bars indicate system inputs; green bars indicate meat and dairy production; and grey bars indicate macronutrient availability at intermediate stages of the chain.

The pathways of macronutrients from crop production to residual food availability are shown for calories, digestible protein and fat in Fig 1A–1C. Across all macronutrients, the relative magnitude of exports, imports and stock variation is small, and approximately balance as inputs and outputs to the food system. This result is in line with India’s orientation towards meeting food demand through self-sufficiency agricultural policies [28, 29]. This study’s scenarios are therefore designed to assess whether this same emphasis on self-sufficiency in food supply through to 2050 could be achieved through waste reduction and crop yield improvements alone.

In 2011, India produced 3159kcal, 72g of digestible protein, and 86g of fat per person per day (pppd) (Fig 1A–1C). Across the system, this resulted in average food availability of 2039kcal, 48g digestible protein, and 49g fat pppd; this represents a loss across the food supply system of 35%, 33%, and 43% in calories, digestible protein, and fat respectively.

Our top-down supply model has been cross-checked against India’s National Sample Survey (NSS) data—this reports nutritional intakes bi-annually measured through national household surveys. In its 68^th^ Round (2011–12) report, the NSS reported average daily intakes of 2206kcal and 2233kcal in urban and rural areas, respectively; 60g of protein in both demographics; and 58g (urban) and 46g (rural) of fat [30]. Our top-down analysis therefore suggests slightly lower caloric availability than NSS intake figures (but with a discrepancy of <10%); and strong correlation regarding fat intake. Since NSS data reports total protein and take no account of quality or digestibility, our results of digestible protein are not directly comparable. However, with digestibility scores removed, our analysis suggests a total average protein availability of 57g pppd—within 5% of NSS intake results.

Despite the acknowledged uncertainties in FAO FBS datasets (see Supplementary discussion), the strong correlation (within 5–10%) between our top-down supply model and reported household intakes (bottom-up approach) gives confidence in the use of FBS data for high-level food chain analyses such as attempted here.

The largest sources of loss identified in the Indian food system for calories and protein lie in the agricultural production and post-harvest waste stages of the chain, with lower but significant losses in processing and distribution. Consumption-phase losses are comparatively small. Higher losses of fat occur predominantly due to the allocation of oilseed crops for non-food uses; this is in contrast to digestible protein where losses to competing non-food uses are negligible.

In contrast to the average global food supply system, the conversion of crop-based animal feed to meat and dairy produce in India appears comparatively efficient, with an input-output ratio close to one for calories and protein, and an apparent small production of fats [31]. It is one of the few agricultural systems in the world where the majority of livestock feed demand is met through crop residues, byproducts and pasture lands—its lactovegetarian preferences tend to favour pasture-fed dairy cattle over grain-fed livestock such as poultry (ibid).

Average per capita supply across all macronutrients falls below average per capita minimum requirements. The magnitude of this issue in India emerges via the population-intake distributions. With extension of average macronutrient availability to availability across the population distribution (using a log-normal distribution with CV of 0.26), 66% (826 million) and 56% (703 million) of the population are at risk of falling below recommended energy and protein requirements, respectively.

### Potential future pathways

#### Scenario results for 2030

Results from scenario analyses for potential food waste reduction and crop yield improvements are summarised in Table 1. Note that we have assumed no change in income/dietary inequalities, hence the CV in distribution has remained constant.

**Table 1:** Mean macronutrient availability in baseline and potential waste and yield scenarios in 2030.

Scenario	Mean caloric availability; kcalpppd (percentage of population below average requirement)	Mean digestible protein availability; gpppd (percentage of population below average requirement)	Mean fat availability; gpppd (percentage of population below average requirement)
Recommended Daily Intake (RDA)	2269	50	
2011 Baseline Scenario	2039 (66%)	48 (56%)	49
2030 Baseline Scenario	1665 (89%)	39 (83%)	40
Scenario 1 (halving food losses)	1754 (84%)	42 (75%)	43
Scenario 2 (achieving 50% AY)	1675 (88%)	40 (81%)	41
Scenario 3 (achieving 75% AY)	1831 (80%)	42 (75%)	46

Average macronutrient availability in baseline and projected scenarios to 2030, relative to average population requirements. Scenario 1 is based on the assumption of halving food losses across the supply chain; and scenarios 2 and 3 achieving 50% and 75% of attainable yields across all crops, respectively. The percentage of the population which would fall below average requirements based on dietary distribution data is reported in brackets.

Under all scenarios, waste or yield improvements fail to keep pace with population growth through to 2030; average per capita caloric, digestible protein and fat availability all fall below the 2011 baseline. Under current levels of dietary inequality, distribution of availability highlights even greater potential malnourishment. The majority (>75%) of the population are at risk of falling below requirements in energy and protein availability in all scenarios. This represents severe malnutrition across India in 2030, even in the case of significant and ambitious yield and efficiency improvements.

Under these scenarios, India would fall far short of reaching the SDG2 target of Zero Hunger by 2030.

#### Scenario results for 2050

**Fig 2:**
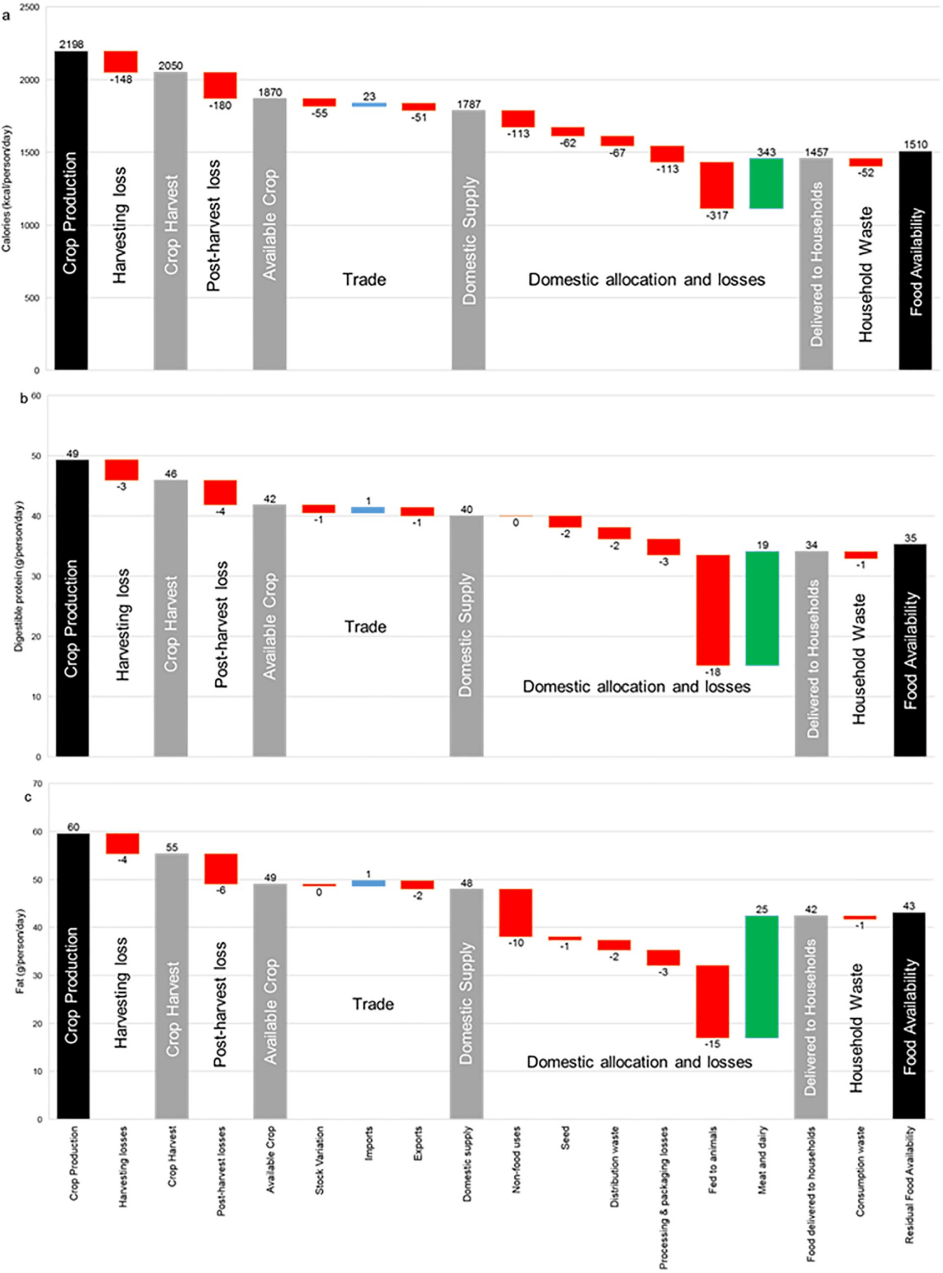
Production and losses in the Indian food system from field to fork under baseline conditions in 2050. Food pathways in (a) calories; (b) digestible protein; and (c) fat from crop production to residual food availability, normalised to average per capita levels assuming equal distribution under 2050 baseline conditions. Red bars (negative numbers) indicate food system losses; blue bars indicate system inputs; green bars indicate meat and dairy production; and grey bars indicate macronutrient availability at intermediate stages of the chain.

India’s anticipated population growth, in addition to potential impacts of climate change on crop yields, could have severe implications on household macronutrient supply by 2050. Our 2050 baseline scenario demonstrates these potential impacts, assuming gains in crop yields were to stagnate at current levels. The full supply chain pathways are shown in Fig 2A–2C. Even at the top level of the supply chain (crop production phase) mean provision per person would fall below average requirements in all macronutrients (2198kcal, 49g protein, and 60g fat per person). Although reducing food system losses plays an important role in improving availability at the household level, this result highlights the necessity of also achieving substantial crop yield improvements at the top of the supply chain.

How these variables impact on availability at the household level in our 2050 baseline, and three scenarios is detailed in Table 2, with baseline distributions provided in Supplementary Fig 1A–1C. As shown, even in the case of scenario 1 (halving of supply chain loss and waste), and scenario 2 (increase to 75% of AY), in 2050 greater than 80% of the population would potentially fall below average requirements in energy and protein. Only in the case of significant yield increases to 90% AY (scenario 3) would projected levels of malnourishment approach current levels. This would still leave 62% and 56% of the population at risk of falling below recommended caloric and protein requirements, respectively.

**Table 2:** Mean macronutrient availability in baseline and potential waste and yield scenarios in 2050.

Scenario	Mean caloric availability; kcalpppd (percentage of population below average requirement)	Mean digestible protein availability; gpppd (percentage of population below average requirement)	Mean fat availability; gpppd (percentage of population below average requirement)
Recommended Daily Intake (RDA)	2269	50	
Baseline 2050	1405 (97%)	33 (95%)	42
Scenario 1 (halving food losses)	1661 (89%)	39 (83%)	51
Scenario 2 (achieving 75% AY)	1721 (86%)	40 (81%)	57
Scenario 3 (achieving 90% AY)	2099 (62%)	48 (56%)	66

Average macronutrient availability in baseline and projected scenarios to 2050, relative to average population requirements. Scenario 1 is based on the assumption of halving food losses across the supply chain; and scenarios 2 and 3 achieving 75% and 90% of attainable yields across all crops, respectively. The percentage of the population which would fall below average requirements based on dietary distribution data is reported in brackets.

## Discussion

Our analysis utilised a framework for evaluation of the whole food system (from crop production through to residual food availability) by normalising to consistent and relatively simplistic metrics (per person per day). This holistic approach is critical for identifying levers within the food system which can be targeted for improvements in food security and efficiency of supply. The basic framework is replicable and could therefore be adapted for analysis of any dietary component (for example, micronutrients or amino acids and at a range of scales (global, regional, or national). This allows for similar analyses to be carried out for any nation, potentially allowing for improved understanding of hotspots in the food system and opportunities for improved efficiency. As such, it could then allow national food strategies to focus on components which are likely to maximise improvements.

Overall, our analyses indicate weaknesses in India’s current reliance on domestic food production. Further calculation, based on FAO FBS, make this explicit: in 2011 India’s population was 17.8% of the global total, yet produced only 10.8%, 9%, and 11.8% of the world’s total calories, digestible protein and fat respectively. Based on calculations using FAOstats global crop production data and nutritional composition factors, in 2011 world crop production totalled 1.34x10^16^kcal; 3.62x10^14^g digestible protein; and 3.33x10^14^g fat. 2011 Indian production amounted to 1.44x10^15^kcal; 3.27x10^13^g digestible protein; and 3.93x10^13^g fat. Even in a highly efficient food system, self-sufficiency is impossible to achieve based on such production levels and the need to provide sufficient nourishment for all. Likewise, even if Indian population figures were to plateau, it is unlikely that domestic production alone would be sufficient to close the current food gap.

Current malnutrition levels—defined here as insufficient macronutrient availability—in India are already high. Sufficient nutrition requires adequate availability and intake of all three macronutrients. Impacts of insufficient protein and energy intake can often be difficult to decouple, and are often termed protein-energy malnourishment (PEM)—PEM has a number of negative consequences including reduced physical and mental development [32]; increased susceptibility to disease and infection; poorer recovery and increased mortality from disease; and lower productivity [33]. Our results indicate that India’s self-sufficiency model—a reliance on domestic crop yield increases and waste reduction strategies—will be insufficient to meet requirements across all three macronutrients. Levels of undersupply and consequent malnutrition would show a significant increase in both 2030 and 2050 scenarios.

This has important implications for forward planning to effectively address malnutrition. Policy incentives in Indian agriculture since the Green Revolution have predominantly been focused on achieving caloric food security through increased production of cereals (wheat and rice) [9]. This has resulted in a heavily carbohydrate-based diet (> 65–70% total energy intake [34]) which may be significantly lacking in adequate diversity for provision of other important nutrients [35]. Widespread lactovegetarian preferences have further reduced the scope for dietary diversity [36].

If trying to address caloric inadequacy alone, efforts to increase output of energy-dense crops (i.e. cereals, roots and tubers) may seem appropriate, and has largely been India’s focus to date [8]. Our analysis, however, strongly suggests the need to shift dietary composition away from reliance on carbohydrates towards a more diversified intake of protein and fats (with diversification also contributing to a reduction in micronutrient deficiency) [37]. Forward planning therefore needs to simultaneously address caloric inadequacy and malnourishment through balanced, increased supply and intake of high-quality proteins and fats.

Our examination of macronutrient supply in India indicates large inequalities in availability across the population. This is likely to be closely coupled to the high levels of income inequality and poverty which remain in India today [8]. Large inequalities in food supply and dietary intake will make it increasingly difficult for India to address its malnutrition challenges; our assessment of potential improvement scenarios highlight that, even in cases where average macronutrient supplies meet requirements, the high CV in distribution still leaves a large proportion of the total population at risk of malnourishment. Whilst the RDA values used in this analysis account for distribution in nutritional requirements of individuals, they do not account for the distribution in intake. To meet SDG2 (whereby *all* individuals’ requirements are met) at current levels of inequality, the national mean intake would therefore have to increase to 3600kcal pppd; 82g pppd digestible protein; and 105g pppd fat. This is well above current national pppd supply values, even if crop production-phase level were to be at the top of the food system.

It should be emphasised that this work is a largely computational, supply-driven analysis exploring the domestic capacity of India’s food. Our results are not intended to imply actual future scenarios of Indian malnutrition. Projections of acute food shortage implied within this analysis would be likely to drive market and policy interventions including enhanced trade, in addition to changes in consumer and producer responses. The interaction between supply and demand-side measures, commodity prices, trade, and governmental policy creates an important feedback loop for food pricing, affordability and production [38]. For example, the estimated reduction in per capita food supply and domestic food shortage would be expected to drive an increase in food prices [39,40]. Rising food prices (as are expected across a number of countries where food demand continues to grow [41]) create a number of producer and consumer impacts, including per capita food expenditure, reduced purchasing power for expensive commodities such as meat and dairy products [42], farmer incentives and agricultural investment. Analysis of the drivers of historical food price volatility and inflation in India suggests that both supply and demand-side factors (and the interaction between) play an important role [40].

The impact of feedbacks such as reduced meat and dairy demand (thereby reducing demands for feed, with further feedbacks on food supply and commodity prices) are not reflected within these scenarios, but will play an important role in determining food system dynamics. The impact of domestic food shortages, agricultural prices and balance within international markets is particularly pronounced in India where the agricultural sector accounts for the employment and income of a large percentage of the population [43]. Literature on the interactions between poverty, agriculture and food prices is extensive; many studies indicate that, since a large share of the world’s poor are rural, high food prices have a positive long-term impact on poverty reduction. However they have negative impacts on poverty and malnutrition in the short-term [39,44–48]. The lack of domestic capacity in India to meet the full nutritional needs (balancing caloric, protein and micronutrient requirements) of its population is likely to increase the demand for commodity imports. This in turn creates further feedbacks on domestic prices, farmer income and inevitably poverty reduction [46]. Further work on the economic dimension to Indian food security—within the context of value chain potential and efficiency evaluations in this study—is therefore crucial to develop better understanding of their interactions and policy responses.

Overall, our results highlighted several key points:

- production quantities at the farm level are very low relative to global average production;
- low import and export values produce an approximately balanced trade model; this correlates with India’s self-sufficiency focused agricultural and food policies;
- harvesting, post-harvest and distribution losses in the supply chain form a large proportion of total food system inefficiencies;
- a moderate amount of energy and fat (but not protein) is allocated to non-food uses, although this is significantly less than global average non-food allocation;
- India’s caloric and protein losses in the conversion of edible crops to livestock are small due to the dominance of pasture-fed livestock such as dairy cattle. The large nutritional gains achieved through increased milk consumption in India suggest this may be a beneficial trade-off in agricultural land for provision of high-quality protein.

Our examination of the food supply chain in India identified harvesting, handling and storage losses, and top-level crop production to be the key intervention phases for improving food security. The approach not only adds value in the identification of ‘hotspots’ of wastage and inefficiency, but also allows for an understanding of the magnitude of change required to produce a certain food supply chain-wide result. Our analysis highlighted that, despite being an important mechanism for improving food security, even a 50% reduction in food loss/waste (a challenge that is achievable but would take significant economic, infrastructural and educational investment) alone would be largely insufficient in ensuring food security in India.

Increased production at the agricultural level must therefore be a focus for both near and long-term food security. The viability of achieving yields close to 75% AY in the near-term (to 2030), across the range of available crops, needs to be more closely considered. For several staple crops, a yield increase upwards of 30% and 50% would be required for attainment of 75% and 90% AY, respectively (see S2 Table). The challenge in reaching close to 90% AY (i.e. almost maximum yield) is substantial; many developed countries have not yet reached such levels [23].

The potential resource limits and environmental implications needed to achieve such yields also need to be given consideration in order to optimise crop selection and mitigate negative impacts. The yield gap could predominantly be closed through improved water and nutrient management [23]. Depleting groundwater resources through agricultural irrigation in India raises key concerns over long-term water security [49][50], and whether water availability is likely to impose a resource limit on yield attainment. Improved yields through increased fertiliser application raise similar sustainability concerns; nitrous oxide (N_2_O) is a key source of greenhouse gas (GHG) emissions, a major source being microbially-mediated emissions as a result of nitrogen fertiliser application to agricultural soils [51]. There may therefore be a significant GHG penalty in closing the current yield gap.

It should be noted that this study has considered only yield improvements through traditional crop varieties. Genetic variation and modification of crop strains may offer further potential for yield increases, in addition to increased resilience to pests, disease and climatic impacts [52]. However, with the exception of Bt Cotton, genetically modified (GM) crop varieties are banned from commercial crop production [53]. Despite the introduction of GM field trials in recent years, they continue to face significant resistance across a range of stakeholder groups [54].

Our analyses for 2050 highlight severe food security challenges for India, even in scenarios which assume attainment of 90% AY for all crops. In addition to the hotspots identified for further focus to achieve near-term improvements, long-term strategies require increased consideration of the impact of potential climatic changes. India’s staple crops–wheat and rice—show particular vulnerability; in the near-term, CO_2_ fertilisation may offer some positive yield impacts, however, simulated climate models suggest this effect is likely to be cancelled out if global mean temperature increase reaches a 3°C threshold in wheat (2°C for rice) [55]. This suggests negative climate impacts may only begin to arise from mid-century onwards. Failure to build capacity and agricultural resilience in the interim could result in severe food deficits should a 2°C or 3°C warming threshold be breached. Planning strategies should therefore not only aim to adapt to gradual near-term impacts of a changing climate, but importantly focus on capacity-building for a resilient food system in a warmer post-2050 world.

Our 2050 scenarios are based on assumptions which are sensitive to change; we have assumed BAU climatic-yield factors, and increased meat and dairy intakes in line with FAO projections. Both of these assumptions could change based on global GHG mitigation progress, and governmental or social interventions on meat consumption. In addition, it is recognised that some potential climatic impacts could be reduced through shifts in crop production regions and seasonal cropping patterns [24]. While such changes may marginally change the scale of the food supply and malnutrition challenge, the overall conclusions remain the same. Climatic and livestock impacts may serve to exacerbate the issue, however, India would continue to face a severe risk of domestic food shortages regardless of these additional pressures.

To deliver effective recommendations for addressing macronutrient undersupply and malnutrition, two key components need to be further explored. Firstly, there needs to be better understanding of optimal crop selections to maximise production and consumer supply of energy, digestible protein and fats alike. This has to be analysed with key resource and environmental constraints in mind to deliver a more optimal and sustainable domestic food system. This should include consideration of options outwith traditional domestic agricultural practice, such as genetic modification, industrial biotechnology and biofortification [56,57].

Secondly, India’s role within global food markets needs to be more closely assessed. To successfully address malnutrition, India will likely have to fill the gap between domestic production and food demand through increased imports. Food imports can have a significant impact on domestic prices, and the dominance of agriculture as a primary source of employment in India may be a negative influence on farmer livelihoods [9]; and further, a large increase in food imports could potentially reduce energy-protein intake for the poorest 30% of the population [46]. This means appropriate economic and social analysis must be carried out to try to optimise import quantities and products which will have minimal domestic impacts. The importance of reducing economic and dietary inequalities makes this even more crucial.

In order to ensure a resilient food system, such analyses and recommendations should be made alongside consideration of potential climatic impacts in the medium- and long-term. This would allow for appropriate choices to be made in the near-term that are also sustainable in a changing climate. The implications of our analysis for health, social, and environmental policy is discussed in detail in our Supplementary Discussion.

Closing its current food supply and nutrition gap while meeting increasing population demand will require a combination of domestic measures to improve agricultural practice and subsequent yields, in addition to a well-planned increase in food imports.

## Supporting information

S1 File Supplementary discussion. (PDF)

S1 Table Loss and waste percentages by food chain stage and commodity group for South and Southeast Asia. Due to poor data availability on India-specific food loss figures, regional average figures from the FAO were applied to derive estimates of macronutrient losses at each stage in the Indian commodity chain.
(PDF)

S2 Table Assumptions and sources for figures used within all scenarios from 2011 baseline to 2050 scenarios. (PDF)

S3 Table Indian baseline and attainable yield (AY) values for key crop types. Year 2000 and all attainable yield values have been derived from Mueller et al. (2012)[23][23][23][23][23](23)(23)(23)(23)(23)(23)(23)(23)(23)(23)(22)(21)(21)(21), and 2011 yield data derived from the FAOstats database (http://faostat.fao.org/beta/en/#home). The necessary percentage increase in yield from 2011 levels to reach each of the AY values has also been shown.
(PDF)

S4 Table Average estimated climatic impacts on Indian crop yields in 2050. Average values have been assumed based on the range of historic studies on yield sensitivities and climatic models within literature review [24]. These models are projected on the basis of a doubling of CO2 from pre-industrial (which is approximately equivalent to a business-as-usual scenario).
(PDF)
