# Supplementary material for: Sustainable food security in India—Domestic production and macronutrient availability
Source: PLoS One. 2018 Mar 23;13(3):e0193766. doi: 10.1371/journal.pone.0193766 (PMC5865708; doi:10.1371/journal.pone.0193766)
Supplement: S1 File — (PDF) [file pone.0193766.s001.pdf]

Sustainable Food Security in India – Domestic Production and Macronutrient Needs

Supplementary Information

Supplementary Discussion on FAO Food Balance Sheets (FBS)

The challenge in developing accurate Food Balance Sheets (FBS) at the national and global level are widely acknowledged and discussed by the FAO (1). The accuracy of FBS is constrained by the completeness and reliability of commodity production and utilization statistics in national records.

The high prevalence of small-holder and subsistence farms in India makes estimates of total production challenging—in this case, completeness of data collection as well as the reliability of farmer reports (farmers often equate production with tax collection) introduce uncertainty to final estimates. Such uncertainty is also present in values of non-food utilizations. Import and export data—which is more meticulously recorded—is likely to be the most accurate of the national statistics recorded in FBS. Issues in agricultural and nutritional data collection in India are described in detail within the FAO's 2030/50 Agricultural Outlook (2).

Food loss and waste figures, especially in countries where small-holder farms and local markets are prevalent, has a high level of uncertainty. To our knowledge, national statistics on supply chain losses and waste in India is not available down to the level of commodity and chain stage breakdown. For this reason, published FAO figures on regional losses for South and Southeast Asia were applied in this study (S1 Table). This introduces further uncertainty to supply chain losses.

Where data within FBS is deemed to be incomplete or inconsistent, judgement from national expert opinion and technical expertise within the FAO is used to provide as reflective coverage as possible. While likely to provide a close approximation, this is rarely 100% accurate.

Nonetheless, the FBS is currently the best available data source for construction and analysis of complete commodity chain analysis. Literature is available based on studies conducted at the household level, however, very few studies attempt to provide coverage of the food chain dynamics from crop production through to human consumption. Without a complete overview of the commodity chain, the impacts of interventions (such as improved food management and storage; trade; reduced allocation of crops to non-food uses; improved crop yields) are almost impossible to assess.

As the FAO notes, food balance sheets “provide an approximate picture of the overall food situation in a country and can be useful for economic and nutritional studies, for preparing development plans and for formulating related projects” (1). In this study, we have therefore relied on FAO datasets in order to construct a high-level overview of the Indian commodity chain to assess its overall capacity to meet the country’s growing nutritional demands at present, in the near-, and long-term. This overview will not be perfect in a statistical sense, however its strong correlation (<5-10% discrepancy) with national household surveys gives confidence that it provides a good approximation of the national food outlook. For its utilisation in this analysis—to inform broad policy focus and assess the potential of supply chain interventions—we therefore deem it to be appropriate.

Improved agricultural, food waste and nutritional reporting would allow for more accurate and reliable estimates to be constructed. Such data collection will provide important in informing

future policy and allowing for forward planning in this sector. It should therefore be an important area of focus for India in the coming years.

Supplementary Discussion on Attainable Yields (AY)

Our scenarios to 2050 are therefore modelled on the basis of closure of the yield gap to 75% and 90% attainable yields (AY). To assess whether these estimates were realistic, necessary growth rates were cross-checked based on historical yield growth rates in India.

Wheat yields in India are growing at approximately 0.9% per annum (non-compounding) from 2009 levels—farm yields (FY) and have shown roughly linear growth at this rate over the last decade (3). To attain 90% AY figures used in this study, yields would have to increase by 36% from 2011 levels, equating to a consistent annual growth rate of 0.9-1.0% to 2050. In other words, India would have to maintain its historic growth rates to 2050 to reach this level. Rice yields have been increasing at an annual non-compounding rate of 1.0-1.1% (3). The yield gap to 90% AY (based on figures used in this study) for rice is smaller than for wheat, at 21% from 2011 levels; this converts to a 0.5-0.6% annual growth rate to 2050. India would therefore not need to maintain its consistent historic growth rate of 1.0-1.1% to reach this level.

While 90% AY figures may be achievable based on historic growth rates, it's important to acknowledge that this improvement would have to be maintained over a further 30-40 years. This raises key concerns given water scarcity and soil fertility constraints. The Green Revolution in India allowed it to achieve impressive increases in agricultural output, primarily through improved nutrient management and irrigation networks. Declining water tables are already a primary concern in India (4)—ever-increasing irrigation demands to maintain historic growth rates are unlikely to be sustained to 2050. Further concern over yield

stagnation globally —in wheat, rice and maize in particular—has been raised in recent years (5). Therefore while estimates vary on levels of attainable yield, figures referenced in this study for maximum 90% AY scenarios are assumed to be potentially realistic but highly ambitious. Note that this study is based on traditional crop varieties and has not included potential genetic variation and modification varieties, which are currently not consented in India (with the exception of Bt Cotton).

Supplementary References

1. Food and Agriculture Organization. Food Balance Sheets Handbook [Internet]. FAOstats. 2001 [cited 2016 Jan 20]. Available from: http://faostat3.fao.org/download/FB/*/E
2. Alexandratos N, Bruinsma J. World agriculture: towards 2030/2050: an FAO perspective. Land use policy. 2012;20(4):375.
3. Fischer T, Byerlee D, Edmeades G. Crop yields and global food security. Aust Cent Int Agric Res. 2014;660.
4. Mekonnen MM, Hoekstra AY. Four billion people facing severe water scarcity. Sci Adv [Internet]. 2016;2(2):e1500323. Available from: http://advances.sciencemag.org/content/2/2/e1500323.abstract
5. Ray DK, Ramankutty N, Mueller ND, West PC, Foley J a. Recent patterns of crop yield growth and stagnation. Nat Commun [Internet]. 2012;3:1293. Available from: http://www.ncbi.nlm.nih.gov/pubmed/23250423
