# Supplementary material for: Sustainable food security in India—Domestic production and macronutrient availability
Source: PLoS One. 2018 Mar 23;13(3):e0193766. doi: 10.1371/journal.pone.0193766 (PMC5865708; doi:10.1371/journal.pone.0193766)
Supplement: S1 Table — Due to poor data availability on India-specific food loss figures, regional average figures from the FAO were applied to derive estimates of macronutrient losses at each stage in the Indian commodity chain. (PDF) [file pone.0193766.s002.pdf]

	Agricultural production	Postharvest handling and storage	Processing and packaging	Distribution	Consumption
Cereals	6%	7%	3.5%	2%	3%
Roots and tubers	6%	19%	10%	11%	3%
Oilseeds and pulses	7%	12%	8%	2%	1%
Fruits and vegetables	15%	9%	25%	10%	7%
Meat	5.1%	0.3%	5%	7%	4%
Fish and seafood	8.2%	6%	9%	15%	2%
Milk	3.5%	6%	2%	10%	1%
