# Supplementary material for: Sustainable food security in India—Domestic production and macronutrient availability
Source: PLoS One. 2018 Mar 23;13(3):e0193766. doi: 10.1371/journal.pone.0193766 (PMC5865708; doi:10.1371/journal.pone.0193766)
Supplement: S2 Table — (PDF) [file pone.0193766.s003.pdf]

Scenario	Assumptions
2011 Baseline Scenario	- Production, imports, exports, stocks, seed, feed, and non-food uses from FAO Food Balance Sheets (http://faostat.fao.org/beta/en/#home).<br>- Production, postharvest, processing, distribution and household waste percentage figures by commodity type from FAO (2011) Global food losses and food waste – Extent, causes and prevention. These factors are provided in Table S3.<br>- Nutritional composition factors based on global average used in FAO Food Balance Sheet Handbook.<br>- 2011 population figures based on UN Population Prospects of 1.2474 billion.<br>- Coefficient variation in caloric, protein and fat of 0.26 based on log-normal distribution from FAO (2014).
2030 Baseline Scenario	- Yield (and food production) stagnates at 2011 levels.<br>- 2030 population figures based on UN Population Prospects of 1.5276 billion.<br>- Coefficient variation in caloric, protein and fat of 0.26 based on log-normal distribution from FAO (2014).
Scenario 1 (2030): Halving food losses	- Percentage losses from production, postharvest, processing and distribution were halved their values in baseline scenario.<br>- Yield (and food production) stagnates at 2011 levels.<br>- 2030 population figures based on UN Population Prospects of 1.5276 billion.<br>- Coefficient variation in caloric, protein and fat of 0.26 based on log-normal distribution from FAO (2014).
Scenario 2 (2030): achieving 50% attainable yields (AY)	- Yields for all commodities assumed 50% of their India-specific attainable yield value from Mueller et al. (2012).<br>- Loss and waste percentages assumed the same as in baseline scenario.<br>- 2030 population figures based on UN Population Prospects of 1.5276 billion.<br>- Coefficient variation in caloric, protein and fat of 0.26 based on log-normal distribution from FAO (2014).
Scenario 3 (2030): achieving 75% attainable yields (AY)	- Yields for all commodities assumed 75% of their India-specific attainable yield value from Mueller et al. (2012).<br>- Loss and waste percentages assumed the same as in baseline scenario. These factors are provided in Table S3.<br>- 2030 population figures based on UN Population Prospects of 1.5276 billion.<br>- Coefficient variation in caloric, protein and fat of 0.26 based on log-normal distribution from FAO (2014).
2050 Baseline Scenario	- Average per capita meat demand increases to 18.3kg and milk to 110 kilograms based on FAO projections (Alexandratos & Bruinsma, 2012). This comprises 3.5kg of bovine meat, 1.2kg mutton & goat meat; 1kg pigmeat; 12.5kg poultry; and 0.8kg other meats). Assumes increased feed demand is met on the basis of increased crop allocation

	rather than pasture in line with livestock-specific protein conversion efficiencies from Herrero et al. (2013).<br>- Climatic impacts on yields is assumed based on literature review of impacts in the result of a doubling in pre-industrial CO_2 levels. Yield impacts are summarised in table S4.<br>- 2050 population figures based on UN Population Prospects of 1.62 billion.<br>- Coefficient variation in caloric, protein and fat of 0.26 based on log-normal distribution from FAO (2014).
Scenario 1 (2050): Halving food losses	- Percentage losses from production, postharvest, processing and distribution were halved their values in baseline scenario.<br>- Average per capita meat demand increases to 18.3kg and milk to 110 kilograms based on FAO projections (Alexandratos & Bruinsma, 2012). This comprises 3.5kg of bovine meat, 1.2kg mutton & goat meat; 1kg pigmeat; 12.5kg poultry; and 0.8kg other meats). Assumes increased feed demand is met on the basis of increased crop allocation rather than pasture in line with livestock-specific protein conversion efficiencies from Herrero et al. (2013).<br>- Climatic impacts on yields is assumed based on literature review of impacts in the result of a doubling in pre-industrial CO_2 levels. Yield impacts are summarised in table S4.<br>- 2050 population figures based on UN Population Prospects of 1.62 billion.<br>- Coefficient variation in caloric, protein and fat of 0.26 based on log-normal distribution from FAO (2014).
Scenario 2 (2050): achieving 75% attainable yields (AY)	- Yields for all commodities assumed 75% of their India-specific attainable yield value from Mueller et al. (2012). These are combined with climatic impacts on yields is assumed based on literature review of impacts in the result of a doubling in pre-industrial CO_2 levels. Yield impacts are summarised in table S4.<br>- Average per capita meat demand increases to 18.3kg and milk to 110 kilograms based on FAO projections (Alexandratos & Bruinsma, 2012). This comprises 3.5kg of bovine meat, 1.2kg mutton & goat meat; 1kg pigmeat; 12.5kg poultry; and 0.8kg other meats). Assumes increased feed demand is met on the basis of increased crop allocation rather than pasture in line with livestock-specific protein conversion efficiencies from Herrero et al. (2013).<br>- Loss and waste percentages assumed the same as in baseline scenario. These factors are provided in Table S3.<br>- 2050 population figures based on UN Population Prospects of 1.62 billion.<br>- Coefficient variation in caloric, protein and fat of 0.26 based on log-normal distribution from FAO (2014).
Scenario 3 (2050): achieving 90% attainable yields (AY)	- Yields for all commodities assumed 90% of their India-specific attainable yield value from Mueller et al. (2012). These are combined with climatic impacts on yields is assumed based on literature review of impacts in the result of a doubling in pre-industrial CO_2 levels. Yield impacts are summarised in table S4.<br>- Average per capita meat demand increases to 18.3kg and milk to 110 kilograms based on FAO projections (Alexandratos & Bruinsma, 2012). This comprises 3.5kg of

	bovine meat, 1.2kg mutton & goat meat; 1kg pigmeat; 12.5kg poultry; and 0.8kg other meats). Assumes increased feed demand is met on the basis of increased crop allocation rather than pasture in line with livestock-specific protein conversion efficiencies from Herrero et al. (2013).<br>- Loss and waste percentages assumed the same as in baseline scenario. These factors are provided in Table S3.<br>- 2050 population figures based on UN Population Prospects of 1.62 billion.<br>- Coefficient variation in caloric, protein and fat of 0.26 based on log-normal distribution from FAO (2014).
