# Supplementary material for: Sustainable food security in India—Domestic production and macronutrient availability
Source: PLoS One. 2018 Mar 23;13(3):e0193766. doi: 10.1371/journal.pone.0193766 (PMC5865708; doi:10.1371/journal.pone.0193766)
Supplement: S3 Table — Year 2000 and all attainable yield values have been derived from Mueller et al. (2012)[23][23][23][23][23](23)(23)(23)(23)(23)(23)(23)(23)(23)(23)(22)(21)(21)(21), and 2011 yield data derived from the FAOstats database (http://faostat.fao.org/beta/en/#home). The necessary percentage increase in yield from 2011 levels to reach each of the AY values has also been shown. (PDF) [file pone.0193766.s004.pdf]

Crop	2000 yield (tha^{-1})	2011 yield (tha^{-1})	50% AY (tha^{-1})	Percentage increase from 2011 to 50% AY	75% AY (tha^{-1})	Percentage increase from 2011 to 75% AY	90% AY (tha^{-1})	Percentage increase from 2011 to 90% AY
Barley	0.79	2.35	2.59	10%	3.88	65%	4.66	97.6%
Cassava	25.63	36.48	27.08	0%	28.89	0%	30.46	0.0%
Groundnut	0.97	1.31	1.02	0%	1.18	0%	1.32	0.7%
Maize	1.54	2.48	1.97	0%	2.64	7%	3.11	25.5%
Millet	0.79	1.19	0.88	0%	1.06	0%	1.2	1.2%
Potato	18.41	22.72	18.85	0%	20.35	0%	21.46	0.00%
Rapeseed	0.94	1.26	0.98	0%	1.08	0%	1.19	0.00%
Rice	2.95	3.59	3.19	0%	3.82	6%	4.33	20.7%
Rye	1.72	1.72	1.78	3%	2.39	39%	2.87	66.9%
Sorghum	0.78	0.95	0.94	0%	1.19	25%	1.39	46.5%
Soybean	0.93	1.20	1.06	0%	1.41	18%	1.67	39.2%
Sugarbeet	36.18	36.18	38.07	5%	48.43	34%	57.48	58.9%
Sugarcane	66.53	69.25	69.68	1%	76.29	10%	85.82	23.9%
Sunflower Seed	0.49	0.71	0.69	0%	0.96	36%	1.13	59.6%
Wheat	2.76	2.99	3.08	3%	3.63	21%	4.07	36.2%
