# Supplementary material for: Sustainable food security in India—Domestic production and macronutrient availability
Source: PLoS One. 2018 Mar 23;13(3):e0193766. doi: 10.1371/journal.pone.0193766 (PMC5865708; doi:10.1371/journal.pone.0193766)
Supplement: S4 Table — Average values have been assumed based on the range of historic studies on yield sensitivities and climatic models within literature review [24]. These models are projected on the basis of a doubling of CO2 from pre-industrial (which is approximately equivalent to a business-as-usual scenario). (PDF) [file pone.0193766.s005.pdf]

Crop	Estimated impact of climate change on yield in 2050
Rice	-7.4%
Wheat	-12.0%
Coarse Cereals	-12.0%
Sorghum	-12.0%
Millet	-4.3%
Maize	-2.5%
Pulses	-12.0%
Chickpeas	-12.0%
Peas	-12.0%
Lentils	-12.0%
Oilseeds	0.5%
Groundnuts	-23.0%
Soybean	-18%
Sugarcane	-10%
Potato	-22%
